# The Impact of Intrusive Rumination on College Students’ Creativity During the COVID-19 Pandemic: The Mediating Effect of Post-traumatic Growth and the Moderating Role of Psychological Resilience

**DOI:** 10.3389/fpsyg.2022.789844

**Published:** 2022-04-11

**Authors:** Yanhua Xu, Jiamin Wu, Qian Li, Wei Zeng, Chujin Wu, Yuhao Yang, Zhihao Chen, Ziying Xu

**Affiliations:** ^1^College of Resource Environment and Tourism, Capital Normal University, Beijing, China; ^2^School of Geography, South China Normal University, Guangzhou, China

**Keywords:** intrusive rumination, creativity, psychological resilience, post-traumatic growth, moderated mediation model, COVID-19

## Abstract

**Purpose:**

College students in the pandemic area are experiencing the problems caused by COVID-19 by themselves or people around them, how to cope with the sudden changes and adjust the psychological stress response, and get experience and grow in the fight against the pandemic is a question worth in-depth discussion. The researchers constructed a mediated regulation model to examine the effects of intrusive rumination on the creativity of college students during the COVID-19 pandemic, as well as the mediating effect of post-traumatic growth and the moderating role of psychological resilience.

**Methods:**

A sample of 475 university students from Guangdong Province, China, were surveyed with the Runco Ideational Behavior Scale, the Event Related Rumination Inventory, the Posttraumatic Growth Inventory, and the Psychological Resilience Scale. SPSS (version 23) and PROCESS (version 3.3) were used for correlation analysis, mediation analysis, and mediated moderation analysis.

**Results:**

(1) Intrusive rumination was positively correlated with post-traumatic growth and creativity but negatively correlated with psychological resilience. Psychological resilience was positively correlated with post-traumatic growth and creativity. Post-traumatic growth and creativity were positively correlated. (2) Post-traumatic growth played a mediating role in the relationship between intrusive rumination and creativity. (3) Psychological resilience moderated the first half of the pathway “intrusive rumination → post-traumatic growth → creativity.”

**Conclusion:**

Intrusive rumination affected creativity directly and also indirectly through post-traumatic growth. At the same time, psychological resilience played a moderating role between intrusive rumination and creativity. The correlation between intrusive rumination and post-traumatic growth was stronger when levels of psychological resilience levels were higher.

## Introduction

The novel coronavirus pneumonia (COVID-19) is the most widespread public health emergency to hit humanity in nearly a century. It has spread on an unprecedented scale among more than 150 countries worldwide (American Psychological Association [APA], 2020; Liu Y. et al., 2020). The rapid emergence, transmission and infection of COVID-19 have not only overwhelmed the global public health and social systems, but also influenced the mental health of people around the world. The governments have taken extreme public health measures in response to it (Xiao et al., 2020; Government of Canada, 2021). While interrupting the spread of the pandemic, however, these measures have had a negative impact on the national social structure and economic activities (Nicola et al., 2020). Restrictive measures such as isolation have affected people’s psychological well-being while producing dramatic changes in their thoughts, psychological responses and emotional reactions to the pandemic (Brooks et al., 2020). COVID-19 is regarded as a traumatic event in a number of studies related to public health and mental health (Castelli et al., 2020; Fawaz and Samaha, 2020; Forte et al., 2020).

Recent studies have shown that under the influence of the COVID-19, the public is generally more psychologically stressed and prone to negative emotions such as anxiety and depression. In China, for example, in the early days of the COVID-19 outbreak, a study showed that more than half of the participants perceived a moderate to severe psychological impact of the COVID-19 outbreak, with about one-third of participants experiencing moderate or even severe anxiety (Wang et al., 2020). In North America, severe psychological problems gradually emerging as COVID-19 spread rapidly. The prevalence of depression and anxiety was 44.1 and 47.2%, respectively, in a Canadian study (Nwachukwu et al., 2020) and 24.4 and 28.2% in an American study (Twenge and Joiner, 2020). In addition, the COVID-19 outbreak also triggered post-traumatic stress symptoms (Jiang et al., 2020), with an Italian study (Forte et al., 2020) showing up to 29.5% of post-traumatic stress disorder (PTSD) and an Irish study (Karatzias et al., 2020) showing that 17% of adults in the general population have experienced PTSD symptoms. Students have often been susceptible to the “psychological pandemic” caused by its emotional and academic stress (Kumar et al., 2019; Ahmed et al., 2020; Huang and Zhao, 2020). Studies have shown that COVID-19 has brought more serious mental-health problems to students, with a prevalence of depression of 23.8% (Chi et al., 2020) and anxiety of 24.9%, among which the proportion of severe anxiety is 90% (Talevi et al., 2020). It is worth discussing how college students in areas affected by the pandemic, who were experiencing the distress caused by COVID-19 themselves or those around them, coped with the sudden changes, adjusted to the psychological stress, and grow up in the fight against the pandemic.

The negative emotions that individuals experience under the influence of COVID-19 may disrupt their original self-perceptions or core beliefs, resulting in cognitive imbalance. The stress caused by this cognitive disequilibrium may promote them to develop appropriate cognitive activities. Under the stress caused by cognitive disequilibrium, certain negative effects are inevitable, but the positive side motivates individuals to form cognitive activities accordingly such as intrusive rumination (Zhou et al., 2015). In the past 20 years, with the rise of positive psychology, researchers have found that although individuals experience a traumatic event accompanied by a stress response, they can also make positive changes in a certain field, thereby achieving growth (Tedeschi and Calhoun, 1996). It is closely related to the process of psychological recovery after a traumatic event, when individuals can achieve positive psychological changes reflecting on their emotions and exploring the causes and consequences of a traumatic event (Dunn et al., 2011). And individuals gain positive growth after encountering an pandemic by thinking about their life environment and social environment after the pandemic (Feng et al., 2021). In this context, researchers have gradually developed a new perspective on stress and trauma research that is oriented toward positive-growth research with a focus on post-traumatic growth (Joseph and Linley, 2006). One study has found a strong link between post-traumatic growth and creativity: post-traumatic growth may lead to enhanced creativity (Liang et al., 2021). Since traumatic events may recur in an individual’s life, creative people are often able to convert challenges into a source of inspiration and motivation for their work. It has also been shown that a high level of psychological resilience can mitigate negative emotional reactions to traumatic events, including COVID-19 (Liu C. H. et al., 2020) and therefore can also help overcome the negative emotional distress caused by the pandemic (Prime et al., 2020).

COVID-19 has had a significant impact on the mental health of college students, and creativity as a prior variable can predict individual mental health or adjustment (Wang et al., 2021). The aims of this paper are therefore to explore the relationship between intrusive rumination and creativity and the mediating role of post-traumatic growth between the two, including mental resilience in the model as a moderator. The definitions of these four variables and the relationships among them are described below.

## Theoretical Foundations and Assumptions

### Intrusive Rumination and Creativity

After experiencing a global traumatic event such as COVID-19, individuals not only experience negative psychological states, but are also prompted to re-examine themselves or reconsider their beliefs, which is regarded as a process of *rumination* due to the repetitive nature of this cognitive process (Moberly and Watkins, 2008; Cann et al., 2011). Rumination is a cognitive response in which individuals repeatedly think about traumatic events and their consequences when experiencing such events (McEvoy et al., 2013); Nolen-Hoeksema (1991) defined ruminative thinking as he tendency of individuals to spontaneously repeat thoughts when experiencing negative stressful events, considering it a form of negative thinking about traumatic events and as a form of self-referral. On this basis, a large body of research has demonstrated that if individuals are in a ruminative state of mind, their psychological condition is more susceptible to the negative effects of traumatic events (Nolen-Hoeksema et al., 2008; Liu et al., 2017), and they are more likely to produce symptoms of depression and anxiety when exposed to negative life experiences (e.g., life challenges, being a victim of bullying) because they tend to be in a negative frame of mind (Marks et al., 2010; Garnefski and Kraaij, 2014). Other researchers have argued that ruminant thinking is the repetition of reflections on relevant events based on understanding the meaning of the event, problem solving, recalling the event, and anticipating the future, a view that reflects the positive side of ruminant thinking. Given that there is no uniform definition of ruminative thinking at this stage, this paper will adopt Cann et al.’s (2011) description of ruminative thinking, which he extends to include intrusive rumination and deliberate rumination, the two dimensions of ruminative thinking, one of which is negative and unconstructive and the other positive and constructive. *Intrusive rumination* is a negative coping attitude: when a traumatic event passively intrudes into an individual’s perception, they react with an evaluative, negative emotional state, a non-constructive perception (Cann et al., 2011). *Positive rumination*, as a constructive cognition, means that individuals consciously reduce the negative impact of traumatic events, actively think positively, face difficulties and actively seek solutions to problems. The widespread and rapid spread of COVID-19 has created a sense of crisis in many people, generating many negative emotions and thoughts that lead to intrusive rumination. In fact, intrusive rumination should receive more attention as an important cognitive process that is closely related to physical and mental health.

The development and creation of innovative persons has been a matter of international academic and educational concern. *Creativity* is an intellectual quality that uses all known information to produce something new, unique, and of value to society or the individual, for a certain purpose (Marks et al., 2010). American psychologist J. P. Guilford coined the term *creativity*, arguing that the process of creating fiction is a process of thought and action (Tedeschi and Calhoun, 1996) and emphasizing that creativity is not a special talent of genius. This led researchers to focus on the exploration of everyday creativity, and their understanding of creative personality shifted from a narrow exploration of top talent in certain fields to a broader exploration of the creative personality of ordinary people (Joseph and Linley, 2006). Creativity is a complex structure that involves both cognition and emotion (Csikszentmihalyi, 1996). Some historiometry studies have pointed out that trauma in childhood may contribute to the development of creative potential, and the frequency and intensity of trauma events can help identify areas of creative achievement.

Previous research has shown that certain forms of rumination contribute to the creative process (Vahle-Hinz et al., 2017). Specifically, the relationship between intrusive rumination and creativity needs to be further investigated. Some studies have shown that intrusive rumination contributes to creative processes. The reason may be that intrusive rumination allow individuals to have more consciou s faculties to attend to generating and refining a creative idea or behavior (Cohen and Ferrari, 2010). For example, Verhaeghen et al. (2005) found that traumatic events, while inevitably creating negative emotions leading to intrusive rumination, can also generate creativity, and that high levels of self-reflection and rumination may contribute to creative work. Richards (1981) believed that professionals in the creative field (such as writing) tended to rumination. Intrusive rumination may allow individuals to have more consciou faculties to attend to generating and refining a creative idea or behavior (Cohen and Ferrari, 2010). Furthermore, ruminant thinking affects different professions differently, with knowledge workers being more concerned with ruminating (Krys et al., 2020). In particular, professionals in creative fields (e.g., writing) tend to ruminate (Tsai et al., 2021). Some studies have also investigated that students’ creativity during an pandemic is positively associated with invasive rumination (Wang et al., 2021). Based on this argument, we propose the following hypothesis.

H1: Intrusive rumination is positively associated with creativity.

### The Mediating Role of Post-traumatic Growth

Research has found that in addition to symptoms of stress, individuals experiencing a traumatic event can also derive benefit and experience growth, including *post-traumatic growth*. Post-traumatic growth is also approached as a popularization text, and some psychologists try to establish the concept of post-traumatic growth from a theoretical or analytical depth. Tedeschi and Calhoun (1996) first introduced the concept of post-traumatic growth in their measurement of the phenomenon of positive change in the psychological aspects of individuals from traumatic events. They proposed that the concept should cover four areas:

(a) The traumatic event was impactful.
(b) In struggling with the traumatic event, the individual experienced a positive process of psychological change.
(c) The individual’s level of functioning in at least one area was higher than before the traumatic event.
(d) Growth and psychological distress coexisted.

The concept of post-traumatic growth has prompted psychological researchers to re-examine psychological stress and trauma from a new perspective, with positive-growth research focusing on post-traumatic growth (Joseph and Linley, 2006). The rapid spread of COVID-19 around the world is similar to a deep existential crisis or traumatic experience, making people suffer from not only the existential threat brought by the pandemic but also from anxiety and panic (Emanuel et al., 2020).

The idea that rumination helps to change individuals’ perceptions and emotions about themselves and the outside world, which can lead to post-traumatic growth, is also reflected in the literature (Cann et al., 2011; Stockton et al., 2011; Lindstrom et al., 2013). Post-traumatic growth emerges from the struggle with the traumatic event, and the cognitive component has a key role in its emergence (Tedeschi and Calhoun, 2004). The level of intrusive rumination positively predicts the level of post-traumatic growth because it is the basis of the cognitive processing that triggers post-traumatic growth and is a catalyst for seeking positive meaning from the traumatic event (Cohen and Ferrari, 2010). According to the above research, intrusive rumination is one of the facilitating cognitive emotional mechanisms of post-traumatic growth. Therefore, we propose a second hypothesis:

H2a: Intrusive rumination is positively correlated with post-traumatic growth.

Traumatic events may have different effects on individual creativity. They may cause negative emotions and have a negative impact on creativity (Benedek et al., 2014). However, other research has suggested that post-traumatic growth can help promote individual creativity development (Forgeard, 2013). For example, when studying the relationship between post-traumatic growth and personality intensity, Peterson et al. (2008) found that creativity as a cognitive level of personality intensity was significantly correlated with post-traumatic growth (*r* = 0.21). In the context of COVID-19, it is also worth exploring how the post-traumatic growth of college students after traumatic events affected their creativity development. Therefore, we propose a third hypothesis:

H2b: The post-traumatic growth of college students is positively correlated with creativity.

Based on the above hypothesis and argument, we proposea the fourth hypothesis:

H2: Post-traumatic growth mediates the positive effects of intrusive rumination on creativity.

### The Moderating Effect of Resilience

People differ in how they deal with stress and trauma, and so their responses to different types of emotional distress caused by traumatic events like the COVID-19 outbreak also vary (Killgore et al., 2020). The American Psychological Association (2016) defined *resilience* as “the process of adapting well in the face of adversity, trauma, tragedy, threat, or other significant adversity” (p. 2). This personality trait enables individuals to withstand and overcome difficulties and adversity, and it plays an important role in overcoming the adverse effects of traumatic events (Southwick et al., 2014). In sports, for example, resilience can help athletes adapt to adverse conditions and overcome difficulties, increasing their chances of success (Hill et al., 2018). Although resilience exists within individuals and is a dynamic adaptive process that is difficult to measure, existing research has shown that individuals with higher levels of resilience achieve better mental-health outcomes (such as for depression, anxiety, and post-traumatic stress disorder) in the wake of adversity and major threats (Osofsky et al., 2011). Studies of bereavement (Bonanno et al., 2002, 2005a) and of the terrorist attack on the World Trade Center in New York City on September 11, 2001 (Bonanno et al., 2005b) provided preliminary evidence of mental resilience in adults. In the face of the increasingly severe global COVID-19 pandemic, strengthening the resilience of the public remains a public-health priority.

College students may have unconsciously engaged in intrusive rumination during COVID-19. and those with higher levels of resilience have been more likely to focus on and process positive information and the positive aspects of their personal experience (Hou and Ng, 2014). Intrusive rumination involves sorting out one’s own emotions and exploring the meaning of the causes and consequences of negative events, which may contribute to the process of psychological recovery (Prati and Pietrantoni, 2009). However, some studies regard resilience and rumination as two inverse factors of stress disorder and used them to explore the factors that affect the recovery of people with post-traumatic stress (Blackburn and Owens, 2016). Resilience is considered as a factor that promotes recovery from stress, while rumination is seen as a negative factor that inhibits recovery. As a result, we propose a fifth hypothesis:

H3a: Intrusive rumination is positively correlated with resilience.

According to previous research, resilience and post-traumatic growth are positively correlated, and the level of psychological resilience can predict the level of post-traumatic growth (Wu et al., 2015). Psychological resilience refers to individual’s positive adaptation to adversity (Luthar, 2006; Manyena, 2006), which is a kind of strategy to deal with life adversity. Individuals with high levels of resilience have higher cognitive flexibility, and they can adjust their cognition after a traumatic event so as to maintain good mental health, so as to successfully deal with traumatic events and achieve personal growth (Hooper et al., 2008). In a daily life, individuals may experience traumatic experiences such as interpersonal violence, death of a loved one, natural disasters, serious industrial or other accidents, war, or terrorism (Furr et al., 2010; Eisenberg and Silver, 2011; Dimitry, 2012; Masten and Narayan, 2012; Osofsky and Osofsky, 2013). Although such experiences are a potential risk to mental health and may lead to post-traumatic stress disorder, among others consequences, most people do not develop the such disorders because of the positive effect of resilience (Karam et al., 2014). COVID-19, as a global traumatic event, needs psychological resilience to deal with, which is related to not only the mental health of individuals during the pandemic, but also individuals getting positive results in negative experiences (Li et al., 2015). Therefore, an in-depth study of the relationship between resilience and post-traumatic growth in college students during the COVID-19 pandemic could be significant. We believe that college students with stronger psychological resilience are more likely to achieve post-traumatic growth, therefore, we propose the following hypothesis:

H3b: Resilience is positively correlated with post-traumatic growth.

Creativity has long been associated with resilient adaptation factors (Luthar, 2003); Metzl and Morrell (2008) integrated two pertinent concepts of resilience and creativity in a general model under one general model in which creativity, as a trait, plays a role within the conceptualization of resilience as a multifactor process. Specific aspects of creativity—namely flexible thinking and originality—appear most pertinent to the process of emotional resilience, and a high level resilience can help individuals maintain a good state in traumatic events, thus providing good conditions for the development of creativity (Metzl, 2009). The relationship between these two variables has been little explored; however, the studies that have reached similar conclusions (Wang et al., 2021). In summary, we propose the following hypothesis:

H3c: Resilience is positively correlated with creativity.

Based on the existing research and the above hypotheses, we further propose that

H3: Resilience may moderate the effects of post-traumatic growth on the relationship between intrusive rumination and creativity.

## Materials and Methods

### Participants and Procedure

The sample for this study was drawn from an independent college in Guangdong Province, China. In order to gather preliminary information before the research protocol was finalized, an exploratory focus-group interview was conducted with students convened by faculty members. To comply with public-health requirements, the focus group was conducted in an online-meeting format. During the meeting, most of the students indicated that they had experienced negative emotions during the COVID-19 outbreak due to various factors. College students in the pandemic areas are experiencing the problems caused by themselves or the surrounding people to Covid-19. How to deal with sudden changes, adjust their psychological stress response, and gain experience and growth in fighting the pandemic is a problem worth in-depth discussion.

A total of 918 students participated in the study by completing an online survey. In China, QR codes are widely used because they carry large amounts of information and are easy and inexpensive to use. Therefore, data for this study were collected with the help of QR codes, mainly by showing the two-dimensional code for the questionnaire during breaks in English classes of students in a certain year of their college program. Before the QR code for the questionnaire was shared, the purpose of this study was explained. After giving consent, students who volunteered to do so filled out the questionnaire. The data collection period was from April 10 to June 15, 2020.

In order to better study the psychological status of students in pandemic areas and to avoid invalid samples influencing the data results, the data were preprocessed before analysis. Questionnaires from students outside Guangdong province and from non-pandemic areas were removed, which reduced the sample size to 475. These remaining participants were all from students in the same year of study and comprised 156 males (36.534%) and 271 females (63.466%).

### Materials

The questionnaire used in this survey has five major components: demographic statistical information, the Event Related Rumination Inventory, the Runco Ideational Behavior Scale) Psychological Resilience Scale, and the Posttraumatic Growth Inventory (see Table 1). The demographic statistical information included the gender, home address, and major of the respondents. To order to adapt the scales developed outside China, back-translation (Brislin, 1970) was used: one researcher first translated the original foreign-language scale into Chinese, then another researcher translate the Chinese-language scale back into the foreign language, finally, the third researcher compares and debugs the original scale and the back-translated scale to eliminate equivalence differences. Through the repetition of these three steps, the Chinese-language scale was finalized. In order to make the results of the study comparable across countries and regions, data inequality is resolved before collection.

**TABLE 1:** Scale description.

Scale	Dimension	Cronbach α
Event-Related Ruminant Inventory (ERRI)	invasive rumination and purposeful rumination	0.913
Runco Ideational Behavior Scale (RIBC)	actual behaviors that clearly reflect an individual’s use of, appreciation of, and skill with ideas	0.938
Psychological Resilience Scale(PRS)	goal focus, emotional control, positive cognition, family support, and interpersonal assistance	0.860
Posttraumatic Growth Inventory(PTGI)	interpersonal relationship, new possibilities, personal strength, spiritual change, and appreciation of life	0.958

#### Event Related Rumination Inventory

The Intrusive Rumination Scale from the Event Related Rumination Inventory (Cann et al., 2011) was used in this study. The scale consists of 10 questions, which are rated on a 4-point Likert scale:(1 = not at all, 2 = occasionally, 3 = often, and 4 = always). The Cronbach’s alpha coefficient of the scale in this study was 0.913.

#### Runco Ideational Behavior Scale

This study used the Runco Ideational Behavior Scale (Runco et al., 2000). This scale is commonly used in the evaluation of creativity. Most items describe actual behaviors that clearly reflect an individual’s use of, appreciation of, and skill with ideas. The scale reflects the creativity of subjects in daily life by assessing their actual overt behavior. The scale consists of 23 self-report items and is rated on a 5-point Likert scale (1 = strong disagreement, 2 = basic disagreement, 3 = uncertainty, 4 = basic agreement, and 5 = strong agreement). In this study, the Cronbach’s alpha coefficient for the scale was 0.938.

#### Psychological Resilience Scale

The Psychological Resilience Scale used in this study was proposed by Hu and Gan (2008) after they had examined the concept of psychological resilience as described by domestic and international scholars (e.g., Connor and Davidson, 2003). The 27-item scale is based on the five dimensions of goal focus, emotional control, positive cognition, family support, and interpersonal assistance. Items are rated on a 5-point Likert scale based on the participant’s perception and degree of identification (Strongly disagree = 1, Disagree = 2, Neutral (Neither agree nor disagree) = 3, Agree = 4, and Strongly agree = 5). The Cronbach’s alpha coefficient of the scale in this study was 0.860.

#### Posttraumatic Growth Inventory

The Posttraumatic Growth Inventory used in this study was first proposed by Tedeschi and Calhoun (1996) and later translated and adapted to conditions in China by Geng et al. (2011). The scale includes the five dimensions of interpersonal relationship, new possibilities, personal strength, spiritual change, and appreciation of life, and there are 21 dimensions in total. The items are rated on a 6-point Likert scale according to the participant’s feelings and level of agreement: 1 = never, 2 = rarely, 3 = a little, 4 = sometimes, 5 = often, and 6 = very often). The Cronbach’s alpha coefficient of the scale in this study was 0.958.

### Data Analysis

For this study, a database was created using SPSS 23.0 statistical software, and the collected questionnaire data were entered for statistical analysis. To prevent possible common method bias, the sample data were first subjected to Harman’s one-way test (Podsakoff et al., 2003). Next, the data were tested for trends in concentration and dispersion through a series of descriptive analyses. This was followed by the calculation of Pearson product moment correlation coefficients between the variables to test the degree of linear correlation between the independent, mediating, moderating, and dependent variables. Finally, the moderated mediation models for intrusive rumination and post-traumatic growth were tested using the process plug-in in SPSS (version 3.3), which was developed by Hayes (2013) specifically for path analysis–based moderated and mediated analyses and their combinations. Indicators of conditional direct and indirect effects were tested using a bias-corrected bootstrapping (*n* = 5,000) with 95% confidence intervals, and the effects were significant when the confidence intervals did not include zero (Cero and Sifers, 2013; Liu et al., 2017).

## Results

### Test of Common Method Bias

We ensured the validity of the data analysis by examining the presence of common method bias (CMB) in the administration of the test using the Harman one-way test. Common Method Bias refers to the possibility that the use of the same data source, the same measurement, or the same measurement environment may lead to artificial covariance between the predictor and effector variables and the formation of an incorrect predictive relationship between them (Podsakoff et al., 2003; Zhou and Long, 2004). Since all data for this study were collected using a self-assessment questionnaire in the same setting and at the same time, common method bias needed to be controlled and tested. The Harman one-way test consisted of principal component factor analysis for 81 items in the questionnaire, all except demographic variables. The results showed that 11 factors had eigenvalues greater than 1. The contribution of the 11 factors to the total variance was 66.181%. The first of these factors accounted for only 24.131%, which is much less than 40%, which is the cut-off value for classifying the size of the common method bias (Li, 2018). Therefore, there was no serious common method bias in this study. In other words, the covariance between the dependent and independent variables was largely due to their nature rather than the measurement methods used in the data-collection process.

### Descriptive Statistics and Correlation Analysis

We examined the means, standard deviations, and correlations of the study variables. As shown in Table 2, intrusive rumination was significantly and positively correlated with creativity (*r* = 0.138, *p* < 0.01) and post-traumatic growth (*r* = 0.103, *p* < 0.05), while it was negatively correlated with psychological resilience (*r* = − 0.265, *p* < 0.01). The creativity of participants was significantly and positively correlated with post-traumatic growth (*r* = 0.406, *p* < 0.01) and psychological resilience (*r* = 0.211, *p* < 0.01). In addition, post-traumatic growth was significantly and positively correlated with psychological resilience (*r* = 0.401, *p* < 0.01). Thus, the results of the correlation analysis initially supported the subsequent mediating-effect test.

**TABLE 2:** Descriptive statistics and correlations among variables.

Variables	Mean	SD	1	2	3	4
(1) IR	1.632	0.477	—			
(2) Creativity	3.246	0.575	0.138**	—		
(3) PTG	3.287	1.009	0.103*	0.406**	—	
(4) PR	3.417	0.460	−0.265**	0.211**	0.401**	—

N = 475. IR, intrusive rumination; PTG, post-traumatic growth; PR, psychological resilience. *p < 0.05. **p < 0.01.

### Analysis of Mediators of Post-traumatic Growth

We used Model 4 of the SPSS Process component to perform multiple regression analysis with intrusive rumination as the independent variable, creativity as the dependent variable, and post-traumatic growth as the mediating variable. As shown in Table 3, intrusive rumination was positively associated with creativity (β = 0.226, SE = 0.024, *p* < 0.001) and significantly predicted creativity, and higher levels of intrusive rumination predicted greater creativity in individual participants. Intrusive rumination positively predicted post-traumatic growth (β = 0.559, SE = 0.093, *p* < 0.001), and post-traumatic growth positively predicted creativity (β = 0.226, SE = 0.024, *p* < 0.001).

**TABLE 3:** Results of testing the moderated mediating effect of post-traumatic growth on creativity.

	On PTG				On creativity			
Predictors	β	SE	*t*	95% CI	β	SE	*t*	95% CI
IR	0.559	0.093	5.995 ***	[0.376,0.742]	0.117	0.051	2.315***	[0.018,0.217]
PTG					0.226	0.024	9.421***	[0.179,0.273]
*R* ^2^	0.249				0.174			
F	44.974***				49.840***			

Analyses conducted using PROCESS model 4 N = 475. IR, intrusive rumination; PTG, post-traumatic growth. **p < 0.01; ***p < 0.001.

We utilized the bootstrap method for testing confidence-interval estimates, and the results indicated that the 95% confidence intervals for the direct and indirect effects of intrusive rumination on creativity did not contain 0. Therefore, the partial-mediating-effect equation model for post-traumatic growth held true, and post-traumatic growth was the mediating variable in the relationship between intrusive rumination and creativity. The direct and indirect effects accounted for 70.659 and 29.341% of the total effect, respectively (see Table 4).

**TABLE 4:** Total effect, direct effect and indirect effect among the variables.

	Effect size	Boot SE	Boot CI lower limit	Boot CI upper limit	Relative effect size
Total effect	0.167	0.055	0.059	0.275	
Direct effect	0.117	0.051	0.018	0.217	70.659%
Indirect effect	0.049	0.027	0.001	0.180	29.341%

### Intermediary Moderation Effect

To test H3, the moderating variable, psychological resilience, was added to the first half of the mediated model and a moderated mediated model analysis was conducted using SPSS Process Model 7. The results showed that psychological resilience was positively associated with post-traumatic growth (β = 1.021, *p* < 0.001) with a 95% confidence interval of [0.839,1.203]. The interaction term between intrusive rumination and psychological resilience on post-traumatic growth reached a significant level (β = 0.466, *p* < 0.001).

**FIGURE 1:**
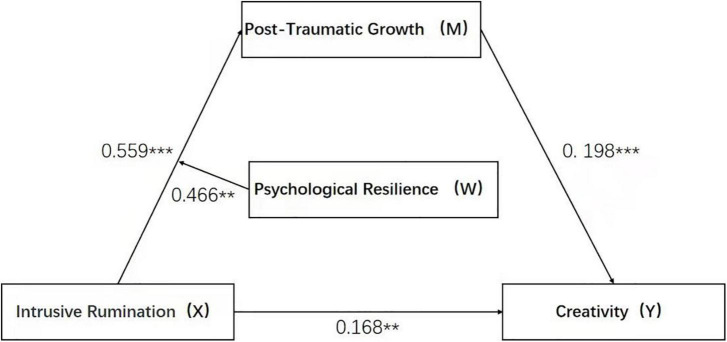
The moderated mediation model. ***p < 0.001, **p < 0.01.

The results showed that proactive rumination was positively correlated with creativity (β = 0.178, *P* < 0.001) with a 95% confidence interval of [0.111, 0.244]. The interaction term between self-efficacy and active rumination had a significant level of effect on creativity (β = −0.186, *P* < 0.01) with a 95% confidence interval of (0.158,0.775) (see Table 5). This model is shown in Figure 1.

**TABLE 5:** Moderated mediation test (*N* = 475).

	On PTG				On creativity			
Predictors	β	SE	*t*	95% CI	β	SE	*t*	95% CI
IR	0.559	0.093	5.995***	[0.376,0.742]	0.117	0.051	2.315*	[0.018,0.217]
PR	1.021	0.093	11.035***	[0.839,1.203]				
PTG					0.226	0.024	9.421***	[0.179,0.273]
IR * PR	0.466	0.157	2.972**	[0.158,0.775]			–	
R^2^	0.223				0.174			
F	44.974***				49.840***			

Analyses conducted using PROCESS model 7 N = 475. IR, intrusive rumination; PTG, post-traumatic growth; PR, psychological resilience. *p < 0.05. **p < 0.01. ***p < 0.001.

To further analyze the moderating effect of psychological resilience, we divided psychological resilience into three groups of high (M + 1SD), medium, and low (M − 1SD) and performed simple slope analysis. The results showed that the 95% confidence intervals of all three groups did not include 0. Psychological resilience influenced the strength of the relationship between intrusive rumination and post-traumatic growth. The moderating effect of psychological resilience in the relationship between intrusive rumination and post-traumatic growth gradually increased from the low group to the high group (see Table 6).

**TABLE 6:** Conditional indirect effect at specific levels of psychological resilience when mediated by post-traumatic growth.

Conditional effect of DR	Effect	Boot SE	95%CI
Low (M − 1SD)	0.345	0.100	[0.149,0.540]
Medium (M)	0.559	0.093	[0.376,0.742]
High (M + 1SD)	0.773	0.134	[0.511,1.036]

## Discussion

### Discussion of the Results

Firstly, this study finds that college students’ intrusive rumination is positively correlated with creativity, which confirms H1 and is consistent with previous studies. Some of the items in RIBS, such as “I am able to think about things intensely for many hours” and “I often have trouble sleeping at night, because so many ideas keep popping into my head,” describe overt behavior similar to rumination which is generally defined means repetitive thought, pondering or meditating on information (Cann et al., 2011; Runco et al., 2000). The process of generating passive and undesired invasive ideas after a highly stressful event can be regarded as intrusive rumination, so the consistency between some items of RIBS and intrusive rumination may lead to a positive correlation between intrusive rumination and creativity. Clinical psychology researchers have studied the relationship between rumination and creativity, and there have been some interesting findings. Different forms of rumination have different mechanisms of action on creativity. Only reflective rumination can positively predict creativity, according to Burwell and Shirk (2007). However, the results of this study match those of many previous studies in which intrusive rumination is closely and positively correlated with creativity. Participating students with high levels of creativity experienced more intrusive rumination during the COVID-19 pandemic, possibly because creativity values free association or fantasy (Wang et al., 2021). Intrusive rumination allows more conscious participation in the production and refinement of a creative idea or behavior (Dijksterhuis and Meurs, 2006). In fact, the relationship between rumination and creativity may vary with the person or environment (Wang et al., 2021). Differently from previous rumination studies, for which data collection took place in a safe environment, data collection for this study occurred in the midst of the COVID-19 pandemic, a traumatic global event. The results of the present study may better reflect reality.

Second, the results confirm H2a, H2b, and H2. Intrusive rumination can positively predict post-traumatic growth. Intrusive rumination increases the negative emotion and cognitive burden of individuals. In order to alleviate the impact of these negative psychological reactions, individuals have to engage in active cognitive processing of trauma-related cues (Ciesla and Roberts, 2007). Therefore, positive interventions in college students’ mental health are of great significance for their post-traumatic growth. Furthermore, post-traumatic growth promotes creativity because the experience of adversity can provide important material, ideas, and motivation for creative projects (Forgeard, 2013). After a traumatic event, it forces us to re-examine our past experiences and perceptions, break down our old worldviews, and construct new meanings and identities. For example, and scientists may seek to understand the reasons for their experience. As well, individuals who have experienced traumatic events and grown as a result can collect materials and ideas in life in a more positive state, thereby stimulating creative behavior. This is the “survivor mission” (Eskreis-Winkler et al., 2014). Therefore, post-traumatic growth can play a mediating role between intrusive rumination and creativity; that is, intrusive rumination can further affect the development of individual creativity after promoting post-traumatic growth.

Third, the research results confirm H3a, H3b, H3c, and previous research results. Intrusive rumination is negatively correlated with resilience. People with high resilience can produce positive emotions and reduce the incidence of intrusive rumination (Li et al., 2018). When individuals generate intrusive rumination because of traumatic events, a higher level of resilience can promote higher cognitive flexibility and better and faster adjustment of emotions. Furthermore, relevant studies have shown that resilience can positively predict post-traumatic growth (Affleck and Tennen, 1996); that is, the higher the level of resilience, the higher the degree of post-traumatic growth, consistent with Hooper et al. (2008), who reported that individuals with high resilience will report higher levels of post-traumatic growth. Individuals with high resilience can better self-regulate and treat events with an optimistic attitude. In order to help themselves out of the plight of trauma, they can maintain an optimistic and positive attitude and fully mobilize the resources of their personality and their environment when facing adversity. In addition, studies have shown that individuals who exhibit resilience or post-traumatic growth can show strong resistance to possible trauma in the future (Davis et al., 2007). Finally, resilience is closely related to creativity, and it can be predicted that college students with higher levels of resilience have more creative thinking (Liang et al., 2021). This is because people with higher levels of cognitive flexibility can better deal with negative situations and can translate negative information into positive ideas, making it easier to recover from adversity and to continue to engage in innovation (McFadden and Basting, 2010). Individuals with a high level of creativity can solve problems in a variety of ways, which is conducive to improving their resilience (Seale et al., 2013). Emotional flexibility is a component of resilience, which is consistent with the flexibility dimension of creative personality (Thompson et al., 2011). Therefore, it is understandable that there is a significant correlation between resilience and creativity.

Fourth, this study found that resilience plays a regulatory role in the process of the intrusive rumination of participating college students affecting post-traumatic growth, which confirms H3. And the higher the level of resilience, the more significant is its regulatory role between intrusive rumination and post-traumatic growth. Post-traumatic growth originates from the struggle with traumatic events rather than traumatic events themselves. In this process, the realization of growth is dependent on individual strength (Casali et al., 2021). When individuals are inevitably immersed in negative thoughts, resilience, as an individual trait, can make individuals recover from and adjust to difficulties, setbacks and misfortunes (Hooper et al., 2008). It can be seen that education should continuously improve the level of resilience of college students, so that even when intrusive rumination occurs after traumatic events, individuals can better restore and regulate their emotions, thereby promoting their continued development.

### Implications

In terms of theoretical significance, this study links intrusive rumination with creativity, expanding knowledge of the impact of intrusive rumination on the creativity of college students in the face of public-health emergencies. Different from previous studies conducted in a safe environment, data collection for this study occurred in the midst of the COVID-19 pandemic, and its results can better reflect reality. In addition, this study analyzed the mediating effect of the post-traumatic growth of participating college students. The results show that intrusive rumination will enhance their post-traumatic growth and ultimately have a positive impact on their creativity, and that resilience has a positive moderating effect on the relationship between intrusive rumination and post-traumatic growth.

With regard to practice, the relationship between the four variables can help teachers and school administrators better cultivate students’ creativity and better attend to students’ mental health after traumatic events. Students inevitably have some negative emotions after experiencing a traumatic event. At such times, they should be encouraged to psychological resilience training. Helping them to look at traumatic events from different perspectives and to discover the potential meaning of traumatic events may reduce their psychological stress and promote their post-traumatic growth, which has positive significance for the cultivation of their creativity.

### Limitations and Future Directions

The limitations of this study are as follows: (1) The study adopts a cross-sectional design. Although a cross-sectional study combined with data analysis can explore the relationship between different variables, it is difficult to determine the causal relationship due to the short time span. In a future study, researchers could conduct repeated surveys of the same respondents over a period of time and expand the coverage of samples.

(2) The data for this study come from self-reports by the respondents. Although there is no serious common method bias according to the Harman single factor test results, the generalizability of the research results is undermined because the respondents are from the same college and are affected by social expectations and their environment. Therefore, in the future, data collection could include parental reports, teacher assessments, and peer nomination methods.

(3) All respondents were from the same university, and the proportion of males and females in the sample is not equal by the limitations of investigation conditions. In future studies, in order to further test the external validity of the research results, more attention should be paid to the extensiveness of sample sources and gender balance in the recruitment of participants.

(4) We have not yet explored the impact of gender, profession, and other factors on the four variables, and control variables could be added to future studies with the goal of furthering understanding of the relationships among the four variables. Future research could also explore the effects of different types of rumination on creativity and post-traumatic growth.

## Conclusion

In this study, a moderated mediation model was constructed to examine the relationship between the intrusive rumination and creativity of college students during the COVID-19 pandemic, the mediating role of post-traumatic growth between the two, and the moderating effect of resilience. The results show that intrusive rumination has a positive predictive effect on creativity, and that post-traumatic growth plays a mediating role between the two. In addition, resilience plays a regulatory role in post-traumatic growth as the first half of the mediating path between intrusive rumination and creativity. Specifically, at a high level of resilience, intrusive rumination has a more significant positive predictive effect on post-traumatic growth. These findings provide more evidence for the relationship between creativity and mental health and reveal the impact of a traumatic event on adolescents.

## Data Availability Statement

The original contributions presented in the study are included in the article/supplementary material, further inquiries can be directed to the corresponding author/s.

## Ethics Statement

The studies involving human participants were reviewed and approved by the Ethics Committee of South China Normal University. The patients/participants provided their written informed consent to participate in this study. Written informed consent was obtained from the individual(s) for the publication of any potentially identifiable images or data included in this article.

## Author Contributions

YX and WZ designed the research. JW, YX, WZ, and QL reviewed the literature and analyzed the data. YX, JW, WZ, QL, CW, YY, ZC, and ZX wrote the manuscript. YX and WZ reviewed and edited the manuscript. All authors have read and agreed to the published version of the manuscript.

## Conflict of Interest

The authors declare that the research was conducted in the absence of any commercial or financial relationships that could be construed as a potential conflict of interest.

## Publisher’s Note

All claims expressed in this article are solely those of the authors and do not necessarily represent those of their affiliated organizations, or those of the publisher, the editors and the reviewers. Any product that may be evaluated in this article, or claim that may be made by its manufacturer, is not guaranteed or endorsed by the publisher.
